# Propensity for selfing varies within a population of hermaphroditic snails: coexistence of selfers, outcrossers and mixed-mating individuals

**DOI:** 10.1098/rsos.230532

**Published:** 2023-10-04

**Authors:** Anja Felmy, Alena B. Streiff, Jukka Jokela

**Affiliations:** ^1^ Department of Biology, Aquatic Ecology Unit, Lund University, 22362 Lund, Sweden; ^2^ Department of Aquatic Ecology, EAWAG, Swiss Federal Institute of Aquatic Science and Technology, 8600 Dübendorf, Switzerland; ^3^ ETH Zurich, D-USYS, Institute of Integrative Biology, 8092 Zürich, Switzerland

**Keywords:** direct selfing rate estimates, mate availability, mixed mating, propensity for selfing, *Radix balthica*, reproductive assurance

## Abstract

To understand mating-system evolution in self-compatible hermaphrodites, variation in selfing rates is highly relevant. Empirical studies are rarely designed to capture variation between individuals, instead often comparing species and populations. Yet, evolution primarily occurs within populations, rendering among-individual variation essential. Observed individual selfing rates depend on the environment (e.g. differences in mate availability) and individuals' propensity for selfing. We quantified individual variation in selfing propensity in the snail *Radix balthica* by conducting laboratory mating trials that manipulated mate availability (low versus moderate) and estimating selfing rates from progeny arrays. We also measured female lifetime fitness. We found substantial among-individual variation in selfing propensity, including pure selfers (32%), pure outcrossers (31%) and mixed-mating individuals that selfed and outcrossed (37%). Experimental levels of mate availability did not significantly affect selfing rates. Selfers had reduced female liftetime fitness. Our results show that the propensity for selfing can differ considerably among individuals, with similar proportions of selfers, outcrossers and mixed maters. As mate availability did not affect selfing, our ‘moderate’ experimental level of mate availability might still have been too low to prompt selfers to outcross. This and the observed fitness differences also cautiously suggest that investigating the heritability of selfing propensities might be worthwhile in this population.

## 1. Background

In its broadest sense, the mating system defines how genes are transmitted from one generation to the next through sexual reproduction [1]. The meaning of the term, however, differs among groups of organisms. In self-compatible hermaphrodites, it mainly describes the relative frequency of self-fertilization (hereafter selfing) versus outcrossing. A frequency of selfing of 20% or lower (selfing rate *s* ≤ 0.2) signifies primary outcrossing, one between 20% and 80% so-called ‘mixed mating’ (0.2 < *s* < 0.8), and one of 80% or higher primary selfing (*s* ≥ 0.8) [2]. Selfing is the most severe form of inbreeding. As such, it increases homozygosity within individuals and may lead to inbreeding depression, with selfed individuals having reduced viability, reduced fertility or failing to thrive altogether if inbreeding depression acts in the early stages of embryo development [3]. The mating system adopted by self-compatible hermaphrodites thus influences the quantity and genetic quality of offspring [1]. Consequently, it has pervasive effects on individual fitness and on the evolutionary and demographic properties of populations and species [3–5].

We can study the mating systems of hermaphrodites by comparing species, populations and individuals. When mating systems have been compared among species, the distribution of selfing rates has been found to be bimodal [6–9]: in both plants and animals, primary outcrossers and primary selfers are more common than mixed maters. In plants, many studies have also documented variability between populations of a single species (e.g. [2,10–12]). For example, in 51% of 105 surveyed plant species, populations fell into several of the three mating-system categories defined above [13]. Our knowledge on animal populations is sparser. Variation among populations was observed in the androdioecious nematode *C. elegans*, whose hermaphrodites cannot mate with each other and where all outcrossing is due to copulations with males [14]. Only few studies have estimated selfing rates or the delay in the onset of reproduction when selfing (termed ‘waiting time’ [15]) across populations of self-compatible hermaphrodites that are capable of outcrossing. In these studies, among-population variation was the norm [16–23]. It is therefore important to characterize species by more than one population-level estimate of the selfing rate, lest crucial differences between populations may be overlooked.

Variation in selfing rates among individuals is equally essential. Evolutionary change primarily occurs within the population; among-individual variation constitutes the raw material natural selection acts upon. If individuals within isolated populations of simultaneous hermaphrodites were uniform in their ability to self and had equal opportunities to outcross, the selection gradient for the selfing rate would be zero: selection can only act on traits that have phenotypic variance [24].

Despite the importance of among-individual variation in selfing rates, empirical studies are not usually designed to capture variation between individuals. Reliable estimates of individual selfing rates require a large number of rather large progenies to be genotyped with molecular markers of sufficient resolution to unequivocally identify outcrossing events [25,26]. Existing studies have shown that selfing rates vary within populations of plants (e.g. [27–29]) and animals (e.g. [18–20,30–34]). However, especially in studies of animals, many estimates rely on small numbers of weakly polymorphic loci, which hampers the detection of outcrossing events, and so may be biased [25,26]. Substantial differences among individuals were also found in proxies of the selfing rate, such as in animals' waiting time [22,35,36] and in the presence of seeds in self-pollinated plants [37,38].

Individual selfing rates likely depend on genetic factors, environmental conditions, and genotype-by-environment interactions. Genetic self-incompatibility systems are well known in plants [39,40] and have recently been discovered in ascidians [41] and oomycetes [42]. Differences in waiting times among families, suggestive of genetic variation in selfing rates, have been found in snails [15,22,35] and flatworms [36]. A key environmental determinant of individual selfing rates is the availability of mating partners. When mates are scarce, selfing provides reproductive assurance. When mates become available, selfing may be avoided because of its potential costs to a selfing individual. These costs include reductions in a selfer's fitness and in the performance of its offspring that are due to inbreeding depression [43,44], but also, for example, an increased vulnerability of selfed progenies to coevolving pathogens and parasites [45]. Yet, to date there are no experimental tests of how individual selfing rates in animals respond to differential mate availability; existing studies [46,47] do not present selfing rate estimates using molecular markers. Here we aim to close this knowledge gap.

It is helpful to think of individual selfing rates within the framework of Brandon's propensity interpretation of fitness [48,49]. Therein an organism's fitness is interpreted as ‘its propensity to survive and reproduce in a particularly specified environment and population’, with the actual survival and reproductive success being just a ‘sometimes reliable—sometimes unreliable—indicator of that ability’ [49, p. 270]. Analogously, an individual's actual (observed, realized) selfing rate can be viewed as an indicator of its propensity to reproduce through self-fertilization in a given environment. Two individuals can have the same propensity for selfing but different actual selfing rates if only one of them encounters environmental conditions (e.g. low mate availability) that trigger the realization of the selfing propensity. Conversely, individuals with different selfing propensities (e.g. high versus intermediate) can have the same actual selfing rate if both experience the same, mate-limited environment. The propensity for selfing thus is a biological property of an individual (the ability and readiness to self) and potentially genetically determined. Its expression, however, depends on the environment. We consider this interpretation helpful because it allows a separate exploration of the individual versus environmental determinants of variation in observed selfing rates, the need for which recent studies have abundantly shown [10,13,23,50].

The freshwater snail *Radix balthica* is an ideal species to study among-individual variation in selfing propensity. Laboratory studies of five Icelandic [51] and one Swiss population [52] have shown that, depending on the population, 23–49% of individuals selfed when prevented from mating. There is also tentative evidence that selfing rates estimated based on molecular genetic markers vary among both populations and individuals in Switzerland [32,33] and Europe [53]. However, as these estimates relied on small numbers of weakly polymorphic allozyme loci [32,33], or relied on microsatellite loci but lacked confidence intervals [53], the occurrence of mixed mating in *R. balthica* cannot yet be confirmed. We previously estimated the selfing rate in the field population studied here using direct progeny arrays and indirect population-level methods based on heterozygote deficiency and identity disequilibria [26]. Both direct and indirect methods showed that the population is almost fully outcrossing in the field [26]. This is in accordance with its fairly high density, here operationally defined as a density where finding two or three snails in close proximity (i.e. on the same stone on a rocky shoreline) is common.

To test how variation in mate availability affects individual selfing rates, we derived individuals' propensity for selfing for two laboratory environments with differential mate availability. We first ascertained how readily 274 laboratory-reared, unmated snails selfed in isolation. We then assigned them to one of two mating treatments, simulating a low or moderate population density. We recorded individual mating behaviour, the production of eggs in isolation (necessarily selfed) and thereafter (potentially outcrossed), the proportion of developing eggs, and the proportion of surviving juveniles. For 56 individuals across both treatments, we estimated individual selfing rates from progeny arrays.

Assuming variation in post-isolation selfing rates, we had two *a priori* predictions: selfing post-isolation is more common under low mate availability (prediction 1) and in snails that selfed in isolation (prediction 2). Prediction 1 arises from the reproductive assurance hypothesis, which posits that selfing enables reproduction under low mate availability but is avoided when outcrossing opportunities are abundant [43,44]. If supported, it suggests that mate availability is a strong environmental determinant of observed selfing rates. Prediction 2 postulates that a period of isolation, followed by a period of access to mates, reveals classes of individuals with different propensity for selfing. Put simply, we expect the isolation phase to identify individuals who *can* self, and the pairing phase to expose those who are also *inclined* to do so. Prediction 2 thus assumes selfing propensities to be stable, fundamental properties, potentially based at least partially in genetic differences. The predictions are not mutually exclusive; environmental and individual (perhaps genetic) factors can jointly shape individual selfing rates. We further explored how variation in selfing propensities is maintained by investigating their association with individuals’ sexual activity, female lifetime reproductive success and their progenies' embryonic development and juvenile survival.

## 2. Methods

### 2.1. Study system

*Radix balthica* is a simultaneously hermaphroditic snail inhabiting the shallow littoral zone of European lakes [53,54]. In Lake Zurich, Switzerland, it exhibits an annual life cycle with non-overlapping generations. Snails hatch from eggs in spring, reach sexual maturity in winter, reproduce from March to May, and then die [55]. During the single breeding season, individuals may copulate repeatedly in both sexual roles and lay hundreds of eggs in distinct egg clutches [55]. They frequently produce a dozen or more clutches across their lifetime [52]. Copulation is unilateral. Lymnaeid snails can store allosperm and should not be allosperm-limited even if they do not mate continuously [56].

### 2.2. Experimental snails

We caught 86 adult snails (P_0_ mothers) at peak breeding season (24 April 2013) in Uerikon, Lake Zurich, Switzerland. In the laboratory at Eawag-Duebendorf, Switzerland (room temperature 18°C), they were kept individually in 200 ml plastic cups filled with aged tap water and fed ad libitum with organic lettuce. Water was changed once a week. In these cups, P_0_ mothers laid egg clutches until they died of natural causes in late spring 2013 (details in [52]). P_0_ mothers were not allowed to mate in the laboratory; their offspring (F_1_ snails) were outcrossed using allosperm stored from copulations in the field, as shown by genotyping P_0_ mothers and F_1_ snails (see Results). Previous work showed that egg clutches collected from the ancestral field population throughout the breeding season often had multiple fathers and contained many unique parental genotypes [55]. This suggests that the fathers of F_1_ snails, while unknown, came from a large and diverse population, and that F_1_ offspring of the same P_0_ mother were often just half siblings. Hence, an overrepresentation of certain fathers among F_1_ snails is unlikely. Each egg clutch laid by P_0_ mothers was placed in a separate water-filled 40 ml plastic cup. After 17 days, when hatching was imminent, each clutch was transferred to a larger 200 ml cup. F_1_ snails were reared in sibling groups and individually from age 19.4 ± 4.1 weeks onwards (mean ± s.d.) to preserve their virginity (details in [52]). This start of isolation is very early; paired control snails only began to mate at age 41.3–45.7 weeks. F_1_ snails were fed *Spirulina* powder mixed with finely ground chalk and flakes of fish food as juveniles, and organic lettuce from age 37.0–42.3 weeks onwards. In spring 2014, mating trials (see next section) were conducted with 274 F_1_ snails derived from 38 P_0_ mothers and 108 sibling groups. The effects of mating trials on mating behaviour, fecundity and the population growth rate are described elsewhere [52]. For a subset of 56 of these 274 F_1_ snails, derived from 22 P_0_ mothers and 38 sibling groups, we estimated selfing rates (see Genetic analysis).

### 2.3. Mating trials

Mating trials began in early May 2014 when F_1_ snails were 52.1 ± 1.0 weeks old. By that time, all F_1_ snails had experienced a prolonged period of isolation of 32.7 ± 3.9 weeks, for the last 6–10 weeks of which they were sexually mature. Accordingly, 38.8% of snails had begun to reproduce through selfing before mating trials began. In the ancestral field population, breeding started in early March [55], and laboratory control snails had started to copulate several weeks earlier (see above).

Two types of mating trials were conducted. In the first type, 137 snails had one mating opportunity with one partner. In the second type, 137 snails had six sequential mating opportunities, one per week, for a total of six weeks. Each mating opportunity involved a different partner, so snails had one partner (first type of mating trial) and six partners in total (second type). All F_1_ snails were subjected to a mating trial; none remained permanently isolated. Pairs of individually marked snails were placed in small, water-filled plastic containers (58 × 38 × 23 mm^3^), with a screen of acrylic glass placed on top to prevent snails from escaping, and a camera mounted 30–40 cm above groups of plastic containers. Mating behaviour was recorded on time-lapse movies (one frame/30 s). During mating opportunities, snails were not fed to ensure optimal visibility of snails on time-lapse movies. A mating opportunity lasted for 10.3 ± 0.8 h, long enough for copulations in both sexual roles, and happened during daylight hours. At the end of a mating opportunity, snails were returned to their individual 200 ml plastic cups filled with water and fresh food. F_1_ snails were euthanized one week after repeatedly paired snails had had their sixth mating opportunity, when mortality began to increase reflecting the snails’ natural, annual life cycle. Additional details of the design of mating trials are provided in [52].

The contrast between once- and six-times-paired snails enables a rigorous test of prediction 1 (selfing post-isolation is more common under low mate availability). Pairing snails once simulated a low population density and a scarcity of mating opportunities, such as in ecologically disturbed or newly established populations. Having just a single mating partner creates substantial obstacles for outcrossing, including an unsuccessful sperm transfer, cryptic female choice, sperm competition with autosperm, and a potential genetic incompatibility between sperm and egg. If a snail's sole mating partner happened to be sterile or incompatible, then this was part of the low-density environment we wanted to simulate. Hence, once-paired snails were clearly mate-limited. By contrast, a choice of six sequential mating partners simulated a moderate population density. We paired them with six partners because in the ancestral field population, egg clutches had 2.1 fathers on average (range: 1–9 [55]). Hence, the average number of mating partners of a free-living snail throughout its life is at least 2.1. We chose to pair snails with six partners because the value of 2.1 likely underestimates the number of potential mates. First, snails can lay dozens of egg clutches, which do not necessarily have the same father(s). Second, not all mate encounters will lead to copulations. Third, if a copulation occurs, there are many obstacles to outcrossing (see above). And fourth, copulations in the male role are not reflected in the paternity of a snail's egg clutches. We paired snails with one partner at a time, rather than with all six partners at once, because sequential matings are more natural than simultaneous ones. First, *R. balthica* does not form aggregations during courtship. Second, although the ancestral population is fairly dense, each stone on the rocky shoreline harbours at most two or three individuals. Third, during the breeding season it is reasonably common to find pairs of snails *in copula*, but exceedingly rare to find clusters of more than two copulating snails.

All except two mating partners were from among the 274 snails, so snails could act both as mothers and fathers. The first mating partner of each snail was size-matched, to ensure that anatomical or developmental differences did not constrain copulation. Mating pairs were set up randomly with respect to relatedness. Consequently, in 22 out of 465 pairings (4.7%) involving 42 F_1_ snails, mating partners had the same P_0_ mother (i.e. full or half siblings). Of these 42 snails, 37 were paired repeatedly, and so were also paired with four to five unrelated snails each, reducing potential effects of mating partner relatedness. The five snails solely paired with a sibling were excluded from all analyses, as was a snail with missing data on female reproductive output. Hence, our sample sizes are 131 once-paired snails (of which we estimated selfing rates for 32) and 137 repeatedly paired snails (of which we estimated selfing rates for 24).

From the time-lapse movies of mating trials, we extracted the number of partners each snail had copulated with as a male and as a female. We also collected all egg clutches F_1_ snails laid both before and after being paired to measure female lifetime reproductive success. We placed clutches in individual 40 ml cups and counted the number of eggs and how many of these contained developed embryos 17 days after oviposition (details in [52]). Then each F_1_ snail's clutches were transferred to two 2 l plastic containers each, where selfed (clutches laid in isolation) and potentially outcrossed (clutches laid post-isolation) F_2_ family groups were reared separately. F_2_ snails were fed a mixture of *Spirulina* algae, finely ground chalk and flakes of fish food.

### 2.4. Genetic analysis

We selected those F_1_ snails (*n* = 56) for selfing rate estimation that post-isolation produced at least 12 F_2_ offspring that reached 12.3 ± 0.5 weeks of age. At that time, some F_2_ juveniles were used in a field experiment and the rest frozen for genetic analysis. As by then 53.4 ± 18.1% of F_2_ offspring born post-isolation had died, we here estimate ‘secondary’ selfing rates after the expression of potential in- or outbreeding depression in early development. For logistic reasons, we did not genotype any juveniles from eggs laid shortly before the experiment was terminated. Hence, the genotyped offspring of repeatedly paired snails originate from eggs laid before mating opportunity 4 (*n* = 6 snails), 5 (*n* = 15 snails) and 6 (*n* = 3 snails), respectively. Offspring of these snails can thus be sired by three to five mating partners.

Of 40 F_1_ snails, 15 or more F_2_ juveniles were available for genotyping; of these we genotyped at least 15, resulting in 16.0 ± 1.9 genotyped juveniles per F_1_ snail (range: 15–22). Of the 16 F_1_ snails with fewer than 15 juveniles available for genotyping, all juveniles were genotyped. This resulted in 8.1 ± 3.6 genotyped juveniles per F_1_ snail (range: 3–14). We also genotyped all 86 P_0_ and 274 F_1_ snails to estimate population-level inbreeding coefficients using Genetix v. 4.05.2 [57] and to ascertain whether F_1_ snails themselves were selfed (electronic supplementary material, table S1). Snails were genotyped for ten highly polymorphic microsatellite loci (GenBank accession nos. KX830983–KX830992) developed specifically for this population by Ecogenics GmbH (Zurich, Switzerland). The genotyping and scoring protocol is described elsewhere [26]. One locus showed a non-negligible frequency of null alleles (locus Rb_3, KX830985) and was excluded from all analyses.

### 2.5. Paternity analysis

Paternity analyses were performed using a custom-built R routine. We excluded 41 juveniles (5.3% of 768 juveniles genotyped) from the analysis because their genotypes did not meet our inclusion criteria. To be included, a juvenile (i) must have at least six loci at which both the juvenile and its mother were genotyped successfully, (ii) could lack a maternal allele at no more than two loci, and (iii) for juveniles with exactly six loci at which both juvenile and mother were successfully genotyped, could lack a maternal allele at just one locus. This left us with 13.0 ± 4.1 successfully genotyped juveniles per family (range: 3–18). For these 727 juveniles, we counted the loci consistent with being outcrossed, i.e. those with one non-maternal allele. We then compared each juvenile's genotype to those of all its candidate fathers, defined as all the potential mates of a juvenile's mother, irrespective of whether copulations had been observed. A candidate father was considered a perfect match if he possessed a juvenile's paternal allele at every locus successfully genotyped in the juvenile, the mother, and himself. Candidate fathers lacking a juvenile's paternal allele at one or several loci were considered non-perfect matches and deemed increasingly unlikely fathers the higher the number of father–offspring mismatches. Additionally, we repeated all paternity analyses using COLONY v. 2.0.5.9 [58] (see electronic supplementary material, Methods), known for its highly accurate parentage assignments provided markers are sufficiently polymorphic [59].

Using these procedures, paternity was assigned to 717 (98.6%) juveniles whose genotypes met the inclusion criteria detailed above. On average, these juveniles had allelic mismatches to their inferred father at 0.15 ± 0.39 loci (maximum at two loci). The assigned juveniles originated from 55 F_1_ snails (13.0 ± 3.9 assigned offspring per F_1_ snail). Paternity assignments were inconclusive for all of the offspring of one once-paired F_1_ snail (see electronic supplementary material, table S2, for numbers of genotyped, successfully genotyped, and assigned juveniles per family). For 705 juveniles, the R routine and COLONY produced identical assignments. Of the remaining twelve juveniles, five were left unassigned by the R routine, four by COLONY, and three were assigned by both but to different fathers; here we gave preference to the R routine (details in electronic supplementary material, table S2). Allelic diversity was high across loci (electronic supplementary material, table S1), yielding powerful paternity analyses. The mean probability of being selfed for offspring identified as selfed was 0.79 ± 0.22, while that of offspring identified as outcrossed was 0.00 ± 0.00 (electronic supplementary material, table S2). Outcrossed offspring were assigned to a father with a probability of 0.93 ± 0.16 (electronic supplementary material, table S2).

### 2.6. Estimation of the propensity for selfing

For individuals with estimated selfing rates (*n* = 55), we ascertained the selfing propensity from their success at selfing in isolation (i.e. the presence/absence of developed embryos produced before the start of mating trials) and the post-isolation selfing rate. We distinguished four groups of individuals. ‘Outcrossers’ neither selfed in isolation nor thereafter. ‘Plastic switchers’ selfed in isolation but switched to outcrossing as soon as they could mate. ‘Plastic mixers’ selfed in isolation yet continued to self at a low rate post-isolation (selfing rate *s* ≤ 0.2). Finally, ‘selfers’ had a high selfing rate throughout their lives (*s* ≥ 0.8). Note that plastic switchers and mixers are two types of mixed maters.

We also ascertained the apparent propensity for selfing for individuals without selfing rate estimates (*n* = 218). We applied three criteria: (i) the production of developed embryos in isolation and (ii) post-isolation, and (iii) the occurrence of at least one copulation in the female role during mating trials, thus allowing for outcrossing. We again distinguished four groups of individuals. ‘Apparent outcrossers' only reproduced post-isolation and mated as a female. ‘Apparent plastic snails’ (i.e. apparent mixed maters) reproduced both before and after being paired and mated as a female. ‘Apparent selfers’ reproduced only in isolation, only after being paired, or during both phases, yet never mated as a female. Finally, ‘female infertile snails' failed to produce any developed embryos; their propensity for selfing remains unknown. These snails were excluded from analyses of selfing propensities.

### 2.7. Statistical analysis

We tested whether the estimated selfing rate of F_1_ snails after the start of mating trials (a proportion) was higher under low mate availability (test of prediction 1) and upon successful selfing in isolation (test of prediction 2) using a GLMM with a beta distribution (*n* = 55). We chose a complementary log–log link function because this improves the model fit in cases like ours, where many proportions are close to one or zero [60]. In beta regression, no observation can equal exactly zero or exactly one, hence selfing rates of zero were replaced with 1 × 10^−7^ and those of one with 0.9999999. Fixed effects were the experimental treatment (once- versus repeatedly paired), the number of developed embryos produced in isolation, and the number of male and female partners snails copulated with. P_0_ mother identity was used as a random intercept to account for potential similarities between siblings that result from having the same mother. As weights we included the number of F_2_ offspring produced post-isolation that were assigned to a father (i.e. the denominator of the selfing rate).

We used eight GLMMs to test if snails with different propensities for selfing differed in their lifetime number of eggs (female LRS) and developed embryos, in their lifetime proportion of undeveloped embryos, and in the proportion of developed embryos that died before reaching the juvenile age of 12.3 ± 0.5 weeks, when surviving F_2_ juveniles were genotyped. We estimated these models for F_1_ snails with successfully estimated selfing rates (four models, *n* = 50; the five outcrossers were excluded due to low sample size) and for all female fertile snails (four models, *n* = 123 or 113, depending on the trait analysed). We fitted GLMMs using negative binomial errors (nbinom 1 family) for the number of eggs and developed embryos, and using Gaussian errors for the proportion of undeveloped embryos and dead juveniles. Despite being proportions, the latter two response variables did not deviate significantly from normality (one-sample Kolmogorov–Smirnov tests: *D* ≥ 0.06, *p* ≥ 0.33). Furthermore, model assumptions were not violated, as shown by both conventional diagnostic plots and those produced by DHARMa, a simulation-based approach to assess the model fit [61]. We also modelled these proportions using binomial errors, including the appropriate weights in each model. While the binomial and Gaussian models yielded similar results, the fit of three out of four binomial models was substantially worse; in the fourth pair of models, the fit was similar. Hence, Gaussian models are shown here, but readers can find the binomial models in the accompanying R Markdown document.

In all eight models, adult body size was used as a continuous covariate, the propensity for selfing as a factor with three levels (‘selfer’, ‘plastic mixer’ and ‘plastic switcher’ when *n* = 50, and ‘apparent selfer’, ‘apparent plastic snail’ and ‘apparent outcrosser’ when *n* = 123 or 113), and P_0_ mother identity as a random intercept. In all models except those of the number of eggs, we also included the ‘pair identity’ on mating opportunity 1 as a random intercept. This accounts for the non-independence of sexual functions within pairs of once-paired snails, where it was, for example, impossible for one snail to remain unmated while its sole mating partner mated. In models of the number of eggs, the selfing rate post-isolation (see above), and the likelihood of mating in both sexual roles among snails with estimated selfing rates (see below), we did not add the pair identity on mating opportunity 1 because doing so resulted in convergence problems. However, of 31 once-paired snails with estimated selfing rate, only six were paired with one another, while 25 were paired with repeatedly paired snails or with once-paired snails whose selfing rate was not estimated. Any non-independence in selfing rate estimates is thus likely small.

Furthermore, we tested if snails that mated in both sexual roles (no/yes) differed in their propensity for selfing using a GLMM with binomial errors and including snails with successfully estimated selfing rates only (*n* = 50, see above). The propensity for selfing (three levels: ‘selfer’, ‘plastic mixer’, ‘plastic switcher’), adult body size (continuous covariate) and P_0_ mother identity (random intercept) were used as predictors.

Finally, we used two-tailed Pearson's *χ*^2^-tests to investigate effects of the experimental treatment of F_1_ snails (once- versus repeatedly paired) on multiple paternity (supporting analysis), selfing (test of prediction 1) and the propensity for selfing (test of prediction 1). Specifically, 2 × 2 contingency tables were used to test for treatment effects on the number of F_2_ progenies with multiple paternity, the number of F_2_ progenies with selfed offspring, and the overall number of selfed F_2_ offspring. Treatment effects on the propensity for selfing in all female fertile F_1_ snails (categories: ‘apparent selfer’, ‘apparent plastic snail’ and ‘apparent outcrosser’) and only in snails with selfing rate estimate (categories: ‘selfer’, ‘plastic mixer’, ‘plastic switcher’ and ‘outcrosser’) were analysed using 3 × 2 and 4 × 2 contingency tables, respectively.

Analyses were performed using R v. 4.2.2 [62]. GLMMs were fitted using ‘glmmTMB’ [63]. The model fit was assessed using ‘DHARMa’ [61]. We tested the significance of random effects with log-likelihood ratio tests (full model versus model without random effect in question). Values are given as mean ± s.d. Scatter plots were prepared using ‘beeswarm’ [64] and depict data points superimposed on boxplots. The entire code and model output are provided as an R Markdown document [65].

## 3. Results

### 3.1. Frequency of selfing and multiple paternity

**Figure 1.**
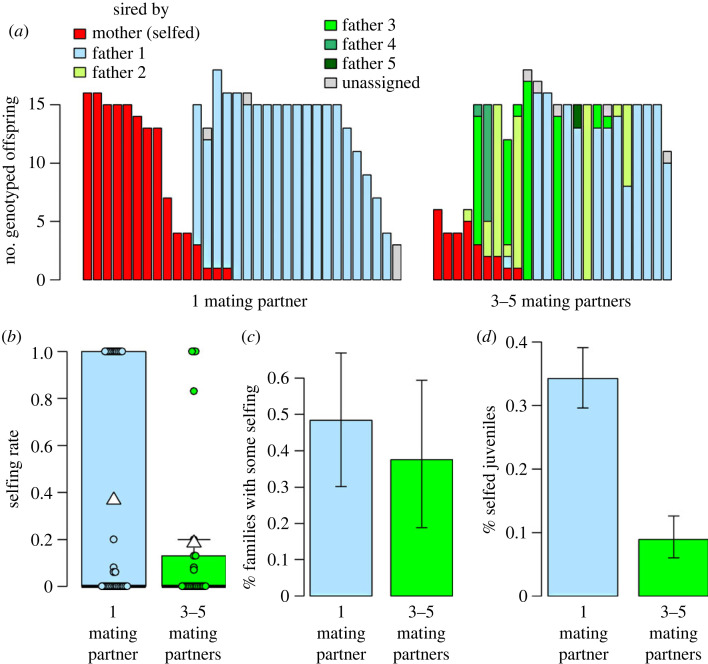
Experimental variation in mate availability did not affect individuals' selfing rates. Snails had either one mating opportunity with one partner, or three to five mating opportunities, each with a different partner. (*a*) Paternity distributions among the progenies of 56 F_1_ snails. Each vertical bar depicts one F_2_ family, with differently coloured sections corresponding to juveniles sired by different fathers. Father 1 refers to F_1_ snails’ first and father 5 to their last mating partner. Identical colours in different F_2_ families do not indicate identical father identities; all in all, F_2_ juveniles were sired by 68 unique fathers, with 10.5 ± 6.4 (mean ± s.d.) juveniles per father (min. = 1, max. = 30). Within pairing treatments, families are arranged according to selfing rate and number of genotyped juveniles. (*b*) Differences in mean selfing rates between once- (*n* = 31) and repeatedly paired snails (*n* = 24) were not statistically significant. White triangles on boxplots show group means. (*c*) The proportion of F_2_ families with non-zero selfing rates was similar among once- and repeatedly paired snails. (*d*) However, once-paired snails produced significantly more selfed juveniles overall. Error bars are 95% confidence intervals [66].

Selfed offspring were found in 43.6% of genotyped F_2_ families (24/55; figure 1*a*). Nine families showed low selfing rates of 20% at most and 15 families high ones of 83% or more. Families with intermediate selfing rates (0.20 < *s* < 0.80) were absent. Overall, 23.2% of offspring were identified as selfed (166/717).

There was no evidence of selfing in either the F_1_ or P_0_ generation. All 274 F_1_ snails were outcrossed, i.e. they possessed a non-maternal allele at one or more loci (mean: 4.6 ± 1.4 loci; electronic supplementary material, table S1). P_0_ mothers of F_1_ snails with genotyped offspring were likely outcrossed too: their genotypes did not show reduced heterozygosity (*F*_IS_ = 0.009, 95% CI: −0.010, 0.068; electronic supplementary material, table S1). P_0_ mother identity explained a significant amount of variance in F_1_ snails' estimated selfing rate post-isolation ($\chi_{1}^{2}=86.17$, *p* < 0.0001).

Multiple paternity was significantly more common among the offspring of repeatedly paired (45.8%) than once-paired snails (12.9%, Pearson's *χ*^2^-test with Yates' continuity correction: observed counts: 4, 27, 11, 13, expected counts: 8.5, 22.5, 6.5, 17.5, $\chi_{1}^{2}=5.83$, *p* = 0.0158; figure 1*a*); note that progenies of once-paired snails show multiple paternity when they contain both selfed and outcrossed offspring. On average, the progenies of repeatedly and once-paired snails had 1.7 ± 0.9 (max. = 4) and 1.1 ± 0.3 (max. = 2) fathers, respectively.

### 3.2. Only subtle effect of mate availability on selfing

**Figure 2.**
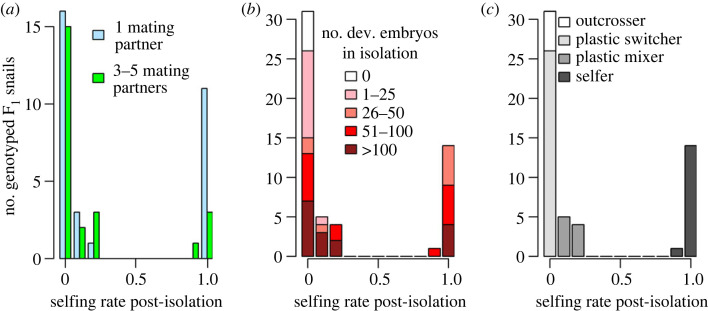
Variation in propensity for selfing among individuals. We ascertained the selfing propensity of snails with offspring that were successfully genotyped and assigned to a father (*n* = 55) based on the presence/absence of selfing in isolation and the post-isolation selfing rate. (*a*) The distribution of post-isolation selfing rates was bimodal among both once- and repeatedly paired snails. (*b*) Selfing in isolation was linked to selfing post-isolation: predominant selfing after mating trials had started only occurred in snails that had selfed successfully in isolation. (*c*) An *a posteriori* categorization revealed four classes of snails with differential selfing propensity: outcrossers that never reproduced via selfing, plastic switchers that selfed in isolation but outcrossed after being paired, plastic mixers that mixed selfing in isolation with a low level of selfing post-isolation, and selfers that selfed fully or predominantly throughout their lives. Plastic switchers and mixers represent two types of mixed-mating individuals. These categories were very similar when based on the number of undeveloped embryos, or undeveloped and developed embryos combined (see electronic supplementary material, figure S1).

Mean selfing rates did not differ significantly among once- (0.37 ± 0.48) and repeatedly paired snails (0.19 ± 0.36; Wilcoxon rank sum test with continuity correction: *W* = 433.5, *p* = 0.25), nor did their variance (Levene's test for homogeneity of variance: *F*_1,53_ = 2.44, *p* = 0.12; figure 1*b*). Accordingly, repeated mating opportunities did not significantly reduce the selfing rate post-isolation (table 1), nor the proportion of families with non-zero selfing rates (once-paired: 48.4%, repeatedly paired: 37.5%, Pearson's Chi-squared test: observed counts: 15, 16, 9, 15, expected counts: 13.5, 17.5, 10.5, 13.5, $\chi_{1}^{2}=0.65$, *p* = 0.42; figure 1*c*). We thus did not find strong support for prediction 1, which was that selfing should be more common at low mate availability, and arose from the reproductive assurance hypothesis. However, as a group, once-paired snails produced significantly more selfed offspring than repeatedly paired snails: 34.2% versus 8.9% (Pearson's *χ*^2^-test: observed counts: 138, 265, 28, 286, expected counts: 93.3, 309.7, 72.7, 241.3, $\chi_{1}^{2}=63.63$, *p* < 0.0001; figure 1*d*). Nevertheless, the distribution of selfing rates was similarly bimodal among both treatment groups (figure 2*a*).

**Table 1.** GLMM on the estimated selfing rate post-isolation. Results are shown for F_1_ snails (*n* = 55) with genotyped offspring that could be assigned to fathers. The response variable was the estimated selfing rate (a proportion) among F_2_ offspring produced by F_1_ snails after the start of mating trials. We used a beta regression model and included the number of F_2_ offspring assigned to a father as weights. Fixed effects were the pairing treatment (once- versus repeatedly; test of prediction 1), the number of developed embryos produced in isolation (test of prediction 2), and the number of male (potential sperm donors) and female partners (potential sperm recipients) a snail copulated with. P_0_ mother identity was included as a random intercept; its effect was significant (*χ*^2^ = 86.17, *p* < 0.0001). Results are provided on a complementary log–log scale. s.e.: standard error.

predictor	estimate (s.e.)	*z*-value	*p*-value
intercept	−0.695 (0.194)	−3.58	0.0003
treatment (once- versus repeatedly paired)	−0.323 (0.264)	−1.22	0.22
no. dev. embryos produced in isolation	0.002 (0.001)	3.35	0.0008
no. male mating partners	−0.130 (0.080)	−1.64	0.10
no. female mating partners	−0.045 (0.053)	−0.85	0.40

### 3.3. Selfing in isolation linked to selfing post-isolation

Predominant selfing after mating trials had started was restricted to snails that had selfed successfully in isolation, while snails without prior selfing exclusively outcrossed post-isolation (figure 2*b*). Consequently, selfing in isolation was associated with a higher selfing rate post-isolation (table 1); this effect remained statistically significant when an extremely prolific snail was excluded (*b* = 0.003, *z* = 2.64, *p* = 0.0083). On average, snails with a non-zero post-isolation selfing rate had produced nearly twice as many developed embryos in isolation (100.0 ± 68.3) as those that exclusively outcrossed after being paired (50.5 ± 59.6). Even though the regression coefficient is very small (0.002 on the complementary log–log link scale; table 1), as it is multiplied by a large number of developed embryos, this effect is not minute. We therefore conclude that our data support prediction 2, which stated that selfing post-isolation should be more common in snails with a history of selfing.

### 3.4. Variation among individuals in propensity for selfing

Snails varied in their propensity for selfing. Of snails with estimated selfing rates (*n* = 55), 9.1% were ‘outcrossers’, 47.3% ‘plastic switchers’, 16.4% ‘plastic mixers’ and 27.3% ‘selfers’ (figure 2*c*). These proportions are based on the production of developed embryos in isolation (and the post-isolation selfing rate), but most outcrossers did not produce any undeveloped embryos in isolation either. Consequently, their proportion (9.1%) was largely unchanged when based on the number of undeveloped (10.9%; electronic supplementary material, figure S1a), or developed and undeveloped embryos (5.5%; electronic supplementary material, figure S1b). Numbers of developed and undeveloped embryos were positively correlated across the whole dataset (Pearson's product-moment correlation: *r* = 0.61, *t*_266_ = 12.53, *p* < 0.0001; electronic supplementary material, figure S1c).

The subsample with estimated selfing rate underrepresents snails that reproduced only before or only after mating trials started (electronic supplementary material, figure S2). To estimate the frequency of selfing propensities more accurately, we thus included all female fertile snails (*n* = 124). This revealed 30.6% apparent outcrossers, 37.1% apparent plastic snails and 32.3% apparent selfers.

The pairing treatment did not influence the selfing propensity of snails with estimated selfing rates (Pearson's *χ*^2^-test: observed counts: 11, 4, 15, 1, 4, 5, 11, 4, expected counts: 8.5, 5.1, 14.7, 2.8, 6.5, 3.9, 11.3, 2.2, $\chi_{3}^{2}=4.98$, *p* = 0.17). However, when using the full dataset, selfing propensities differed among experimental groups (observed counts: 29, 26, 11, 11, 20, 27, expected counts: 21.3, 24.5, 20.2, 18.7, 21.5, 17.8, $\chi_{2}^{2}=15.17$, *p* = 0.0005). Low mate availability led to more apparent selfers (43.9% versus 19.0%) and fewer apparent outcrossers (16.7% versus 46.6%) yet did not affect apparent plastic snails (39.4% versus 34.5%), lending some support to prediction 1.

### 3.5. Reduced female LRS and increased offspring mortality in selfers

**Figure 3.**
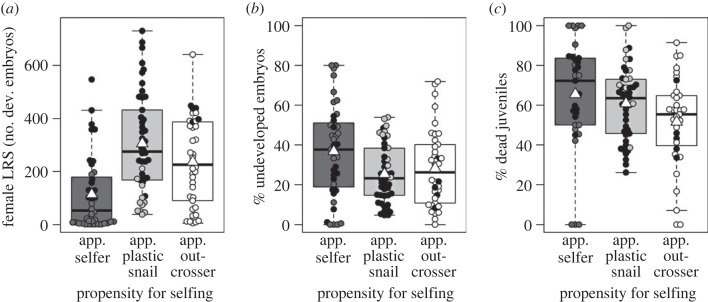
Reduced female LRS and increased offspring mortality in preferential selfers. Individuals with a strong propensity for selfing had the fewest developed embryos (*a*, *n* = 123), the highest proportion of embryos that failed to develop (*b*, *n* = 123), and the highest mortality rate among their juvenile offspring (*c*, *n* = 113). Shown are snails with (black circles) and without genotyped offspring (open circles). We ascertained the apparent propensity for selfing of snails without genotyped offspring based on the timing of egg production and the mating behaviour. Apparent plastic snails represent apparent mixed maters. White triangles on boxplots show group means.

Selfing was associated with a significant decrease in female LRS (number of developed embryos) of 48.9% compared to plastic mixers and of 26.8% compared to plastic switchers (electronic supplementary material, figure S3a, table S3). On the one hand, this was because selfers produced significantly fewer eggs—39.9% and 19.1% fewer than plastic mixers and switchers, respectively (electronic supplementary material, table S3). On the other hand, selfers laid more eggs that failed to develop (28.3%) than plastic mixers (16.2%); the difference to plastic switchers (21.4%) was non-significant (electronic supplementary material, figure S3b, table S3). Outcrossers were excluded because of low sample size (*n* = 5). Across all female fertile snails, female LRS in apparent selfers was not only reduced compared to apparent plastic snails (by 62.9%), but also compared to apparent outcrossers (by 52.0%; figure 3*a* and table 2). Again, this was due to both a reduced fecundity (reductions by 57.0% and 44.9%, respectively) and increased proportion of undeveloped embryos in apparent selfers (36.8% versus 25.4% and 28.2%, respectively; figure 3*b* and table 2).

**Table 2.** GLMMs on female LRS, its components, and juvenile mortality. Results are shown for four models using all female fertile F_1_ snails. Response variables were the lifetime number of developed embryos (female LRS) and eggs, the lifetime proportion of undeveloped embryos, and the proportion of developed embryos that died before they could be genotyped (i.e. before reaching the juvenile age of 12.3 ± 0.5 weeks). For response variables 1 and 2, negative binomial errors were fitted, and results are provided on the log scale. For the two response variables that are proportions, Gaussian errors were fitted, as they did not deviate significantly from normality and as model assumptions were fulfilled. Fixed effects were propensity for selfing (reference level: apparent selfers) and body size (shell length). As random intercepts we included P_0_ mother identity (significant: *χ*^2^ ≥ 5.59, *p* ≤ 0.0181, except for % dead juveniles: *χ*^2^ = 0, *p* = 1) and, in all models except that of the number of eggs, pair identity on mating opportunity 1 (non-significant: *χ*^2^ ≤ 2.51, *p* ≥ 0.11). Although there were 124 female fertile F_1_ snails, sample size in models was reduced because one snail lacked data on body size and ten snails lacked developed embryos that were reared to the juvenile age. Results were very similar when computed for snails with estimated selfing rates only (electronic supplementary material, table S3). s.e., standard error; PS, propensity for selfing; app., apparent.

response	sample size	predictor	estimate (s.e.)	*z*-value	*p*-value
no. dev. embryos (LRS)	123	intercept	2.59 (0.63)	4.11	<0.0001
		PS (app. plastic snail)	1.08 (0.18)	6.13	<0.0001
		PS (app. outcrosser)	0.89 (0.19)	4.81	<0.0001
		body size	0.14 (0.04)	3.27	0.0011
no. eggs	123	intercept	2.75 (0.59)	4.64	<0.0001
		PS (app. plastic snail)	1.02 (0.17)	6.11	<0.0001
		PS (app. outcrosser)	0.83 (0.17)	4.79	<0.0001
		body size	0.15 (0.04)	3.81	0.0001
% undev. embryos	123	intercept	30.78 (14.87)	2.07	0.0384
		PS (app. plastic snail)	−12.77 (3.80)	−3.36	0.0008
		PS (app. outcrosser)	−11.14 (3.92)	−2.84	0.0045
		body size	0.47 (1.07)	0.44	0.66
% dead juveniles	113	intercept	106.09 (19.51)	5.44	<0.0001
		PS (app. plastic snail)	−7.80 (4.94)	−1.58	0.11
		PS (app. outcrosser)	−16.69 (5.12)	−3.26	0.0011
		body size	−2.71 (1.38)	−1.96	0.0496

Additionally, selfing resulted in inbreeding depression in juvenile survival. In snails with selfing rate estimates, the proportion of dead F_2_ juveniles was significantly higher among the progenies of selfers (69.5%) than among those of plastic switchers (53.4%); the difference to plastic mixers (64.5%) was non-significant (electronic supplementary material, figure S2c, table S3). Within all female fertile snails, apparent selfers had more dead juveniles (65.5%) than apparent outcrossers (51.6%), but not significantly more than apparent plastic snails (60.9%; figure 3*c* and table 2).

These results were all corrected for effects of body size, P_0_ mother identity, and, where possible, pair identity on mating opportunity 1 (table 2; electronic supplementary material, table S3).

### 3.6. Mating as a female did not prevent selfing

A strong propensity for selfing was not linked to a lack of sexual activity. Neither the number of male (potential sperm donors) nor the number of female mating partners (potential sperm recipients) was significantly associated with the selfing rate post-isolation (table 1). Many selfers (53.3%) copulated in the female role and so might have received allosperm. Four out of five selfers (80.0%) mated as a male, potentially siring outcrossed offspring. Of snails that selfed post-isolation, 70.8% mated as a female.

**Figure 4.**
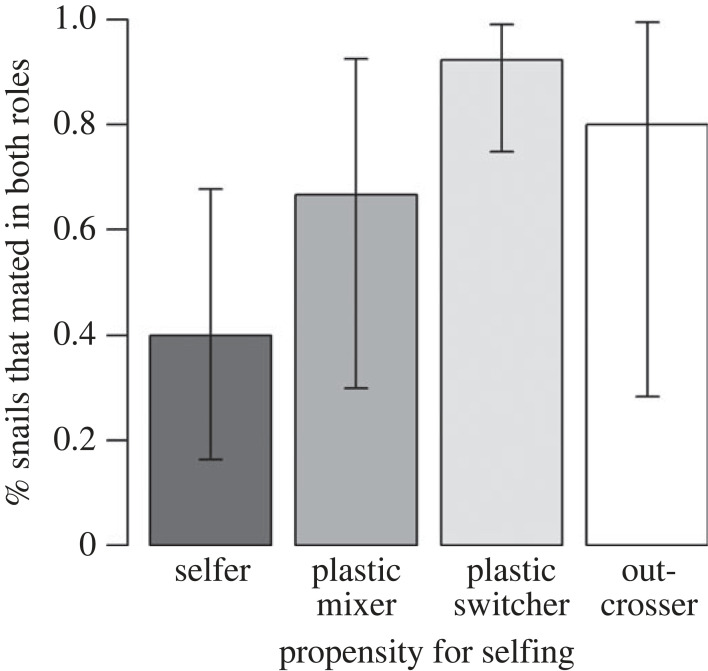
Poor correspondence between propensity for selfing and sexual activity. Although selfers (*n* = 15) were less likely to mate in both sexual roles than plastic switchers (*n* = 26), they showed considerable sexual activity, with 53.3% mating as a female and 80.0% as a male. Plastic mixers and switchers represent two types of mixed-mating individuals. Error bars are 95% confidence intervals [66].

However, the selfing propensity and sexual activity were not entirely independent. Significantly fewer selfers (40.0%) than plastic switchers (92.3%) copulated in both sexual roles (*b* = 2.88, *z* = 3.18, *p* = 0.0015; figure 4); the difference to plastic mixers was non-significant (66.7%, *b* = 1.12, *z* = 1.26, *p* = 0.21). Outcrossers (80.0%) were excluded from the model due to low sample size (*n* = 5; figure 4). Body size (*b* = −0.05, *z* = −0.24, *p* = 0.81) and P_0_ mother identity ($\chi_{1}^{2}=0.0$, *p* = 1.00) did not affect the likelihood of mating in both sexual roles.

## 4. Discussion

We found that propensities for selfing differed substantially between the individuals of a single snail population. Variation among individuals in selfing propensity has not received much attention in the mating-system literature, which has traditionally been focused on comparing species [6–9] or populations [11,13].

We controlled for several possible sources of error that could hamper the estimation of individual selfing propensities. Focal individuals were outcrossed, sexually mature and paired with at least one size-matched partner that was not a close relative. The pairing treatments contrasted two biologically meaningful levels of mate availability. We estimated individual selfing rates post-isolation from progeny arrays, which are almost free of problematic assumptions [26,67,68]. Our molecular markers were highly polymorphic, yielding reliable paternity assignments with sufficient statistical power [26]. Nonetheless, it is important to note that we estimated ‘secondary’ selfing rates, which might be biased if there is in- or outbreeding depression prior to sampling. Here, we found inbreeding depression: juvenile mortality was highest when progenies were selfed, intermediate when mixed and lowest when outcrossed. Most pre-sampling mortality thus likely affected selfed juveniles. Accordingly, there should be no bias in high selfing rates, while low selfing rates (0 < *s* < 0.21) and those of zero might be slightly too low. This does not change the high prevalence of self-fertility in our population (in fact, it underestimates it), nor the bimodal distribution of selfing rates, nor the substantial variation in selfing propensities among individuals.

The mating-system variation was cryptic in two ways. First, among-individual differences in selfing propensities only became apparent in the laboratory. By contrast, both direct and indirect selfing rate estimates of the ancestral field population were near zero across three years and throughout the breeding season [26]. Using the field results only, the population would have been deemed outcrossing and its considerable potential for selfing missed. Second, much variation would have been overlooked had we only recorded selfing during an isolation treatment, without estimating post-isolation selfing rates. This approach, used in pioneering earlier studies (e.g. [69,70]), merely reveals individuals' ability to self but not their inclination to do so, which must be assessed in the face of outcrossing opportunities and using genetic markers. Here we show just how variable the propensity is, comprising selfers, outcrossers, and two types of mixed maters that differ in their inclination to selfing (higher in plastic mixers than in plastic switchers). Selfers (32%), outcrossers (31%) and mixed maters (37%) were approximately equally common. For *Radix balthica*, this confirms the occurrence of mixed mating in a laboratory setting. Selfing-outcrossing thus is a gradient, not a dichotomy—also, and perhaps especially, when studying individuals.

We found selfing propensities to be largely stable properties of individuals: the better an individual was at selfing in isolation, the more likely it was to self post-isolation (prediction 2). In fact, those individuals that did not self while isolated invariably outcrossed thereafter. These pure outcrossers were numerous, representing nearly a third of female fertile snails. Notably, most of them did not produce any undeveloped embryos in isolation either, showing that they did not try to self and failed (perhaps due to early-acting inbreeding depression), but rather did not try at all. Furthermore, given the deliberately late start of mating trials, it is unlikely that many outcrossers would have begun to self if isolated even longer. These results cautiously hint at a genetic background to selfing propensities, which is in line with findings in other hermaphroditic animals [15,22,35,36,41]. An aversion to selfing so strong as to risk reproductive failure when deprived of mates is so clearly maladaptive that a genetic self-incompatibility mechanism appears at least plausible. While common in plants [39,40], such mechanisms have only recently been discovered in animals [41]. To be clear, our study did not estimate the heritability of selfing propensities. We cannot exclude that non-genetic factors caused the observed variation in selfing propensities. Our results merely imply that a detailed investigation of the genetic basis of variation in self-compatibility might be worthwhile in this population.

By contrast, we found only limited support for the importance of mate availability as an environmental determinant of observed selfing rates: selfing post-isolation was not substantially more common under low than under moderate mate availability (prediction 1), as would be expected under the reproductive assurance hypothesis. While selfing was indeed most prevalent when snails were isolated, it often persisted post-isolation. Moreover, pairing snails repeatedly rather than once had a surprisingly subtle effect, significantly affecting neither the mean selfing rate nor the proportion of selfing individuals. This was despite large effect sizes and sufficient statistical power: doubling our sample size would not have rendered any of these effects significant (see R Markdown document). Instead, treatment effects were non-significant because variation among snails was large even within treatment groups. There were only two significant consequences of pairing snails repeatedly. One was a lower overall number of selfed offspring, showing that increased mate availability reduced selfing in high-fitness individuals (i.e. in individuals with at least 15 successfully genotyped juveniles; figure 1*a*). The other was an overabundance of apparent outcrossers and scarcity of apparent selfers when considering the full dataset.

Superficially, the subtlety of these effects contradicts ecological models of selfing, which assume an advantage and thus higher prevalence of selfing at low population density [43,44]. So far, empirical evidence for reproductive assurance in animals is mixed. While some support was found in androdioecious species such as the clam shrimp *Eulimnadia texana* [71] and the nematode *C. elegans* [72], studies of hermaphroditic snails struggled to find any. Selfing rates were not increased in low-density, disturbed or temporary habitats [16,19,21,31], neither the frequency nor duration of outcrossing depended on the length of a mating opportunity [46], and individuals did not adjust their waiting time to the perceived density of conspecifics [47]. Despite these negative results, it is difficult to imagine that the capacity for independent reproduction does not rank among the primary evolutionary drivers of selfing.

Our study offers a potential reconciliation between the reproductive assurance hypothesis and its limited empirical support in animals. First, reproductive assurance matters, but only in extreme situations. Consequently, there is an ascertainment bias: if a population is dense enough to be sampled, it might be too dense for selfing to be common. Second, it is important to understand that a population's observed selfing rate depends on the distribution of individual selfing propensities among its members. As selfing propensities are biological (perhaps genetic) properties of individuals but depend in their expression on environmental aspects (e.g. population density), the relationship between density and population-level selfing rates is complicated. It depends on the relative frequency of individuals that can respond to density variation with increased or decreased selfing, i.e. on the frequency of mixed maters (the plastic mixers and switchers) and selfers compared to outcrossers. Hence, population-level selfing rates can be low because the population mainly consists of outcrossers, or because density is high enough for outcrossing to occur even in mixed maters and selfers. Interpreting high population-level selfing rates is perhaps easier, as they necessitate a sizeable proportion of self-fertile individuals. However, also in highly selfing populations density is difficult to predict because the threshold density beneath which selfing occurs may be population- or species-specific.

These considerations might explain the apparent conflict between our field (near-zero selfing rate [26]) and laboratory results (widespread self-fertility). We think it likely that the high density of our field population prompts even selfers to outcross. Consequently, the evident self-fertility of large parts of the population does not manifest under the current environmental conditions. Interestingly, the commonness of selfing in the laboratory also among repeatedly paired snails suggests that, compared to their free-living conspecifics, these snails were still mate-limited. If so, then a future experiment with massively increased mate availability should reduce selfing to zero.

What, therefore, maintains self-compatibility alleles in our study population, and why have they not gone to fixation? The cost of lacking self-compatibility is obvious—an occasional crash of population size will quickly eliminate any individuals without self-compatibility alleles. If we assume that outcrossers in our study did not self because they lacked self-compatibility alleles (something that remains to be seen), then the benefits of lacking them include a higher female LRS, resulting from increased fecundity and decreased embryo mortality. Also juvenile mortality was lower among outcrossed progenies. Hence, selfing did not only lead to inbreeding depression in embryo development, but also reduced juvenile survival. The presence of inbreeding depression in laboratory settings tallies with findings in other populations of *R. balthica* [73] and in a close relative [69,70], and might represent a major obstacle for the spread of self-compatibility alleles. The reduced female lifetime fecundity of selfers (45% fewer eggs than outcrossers) is interesting as well. We previously found that selfed egg clutches were only half the size of (supposedly outcrossed) clutches laid after a female mating [52]. This study shows that potential compensation mechanisms of selfers, such as a faster clutch-laying rate, were—if at all present—clearly insufficient. Another benefit of lacking self-compatibility alleles might be an increased male LRS, particularly if selfers have sperm discounting [74] or reduced energy allocation to the male function [75]. However, the strong sexual activity of selfers documented here (80% mated in the male role) suggests that reductions in siring success when selfing might be more modest. The ability to self while also acting as a paternal parent is a main requirement of another hypothesis for the evolution of selfing: the 50% transmission advantage of a selfer's genes over those of an outcrosser [43,76]. If future work finds this ability to be common, then it provides a mechanism for maintaining self-compatibility alleles also at high population density.

Finally, it is worth mentioning the dangers of assuming that pairing individuals results in their offspring being outcrossed, as done in some early work (e.g. [69,70]). In our study, 44% of paired snails selfed at least partially, and 71% of snails that selfed post-isolation copulated as a female. Hence, outcrossing is neither guaranteed by pairing individuals once or repeatedly, nor by observing female copulations. Potential reasons for a failure to outcross include, for example, unsuccessful sperm transfer, cryptic female choice, sperm competition with autosperm, and genetic incompatibility between sperm and egg. Hence, to verify outcrossing events, genetic paternity analysis is unavoidable. Risks of failing to do so include underestimating the frequency and extent of selfing, and underestimating the strength of inbreeding depression in studies that compare the offspring of isolated parents (obligately selfed) with those of paired parents (purportedly outcrossed though potentially selfed; e.g. [50,77–79]). We here add to a growing number of studies that tested the assumption of (near-)exclusive outcrossing after pairing empirically, and found it occasionally confirmed [80], yet more often refuted [34,56,81,82].

## Data Availability

Microsatellite markers: GenBank accession nos. KX830983–KX830992. Data available from the Dryad Digital Repository: https://doi.org/10.5061/dryad.xwdbrv1k3 [83]. Additional information, and all R code and model output, are provided in electronic supplementary material [84].
